# Dioleoylphosphatidylglycerol Accelerates Corneal Epithelial Wound Healing

**DOI:** 10.1167/iovs.61.3.29

**Published:** 2020-03-18

**Authors:** Wendy B. Bollag, Lawrence O. Olala, Ding Xie, Xiaowen Lu, Haixia Qin, Vivek Choudhary, Rachana Patel, David Bogorad, Amy Estes, Mitchell Watsky

**Affiliations:** 1 Charlie Norwood VA Medical Center, Augusta, Georgia, United States; 2 Department of Physiology, Medical College of Georgia at Augusta University, Augusta, Georgia, United States; 3 Department of Medicine, Medical College of Georgia at Augusta University, Augusta, Georgia, United States; 4 Department of Cellular Biology and Anatomy, Medical College of Georgia at Augusta University, Augusta, Georgia, United States; 5 Hospital Internal Medicine, Mayo Clinic, Rochester, Minnesota, United States; 6 Department of Ophthalmology, Medical College of Georgia at Augusta University, Augusta, Georgia, United States

**Keywords:** aquaporin 3, phospholipase D2, phosphatidylglycerol

## Abstract

**Purpose:**

In contact with the external environment, the cornea can easily be injured. Although corneal wounds generally heal rapidly, the pain and increased risk of infection associated with a damaged cornea, as well as the impaired healing observed in some individuals, emphasize the need for novel treatments to accelerate corneal healing. We previously demonstrated in epidermal keratinocytes that the glycerol channel aquaporin-3 (AQP3) interacts with phospholipase D2 (PLD2) to produce the signaling phospholipid phosphatidylglycerol (PG), which has been shown to accelerate skin wound healing in vivo. We hypothesized that the same signaling pathway might be operational in corneal epithelial cells.

**Methods:**

We used co-immunoprecipitation, immunohistochemistry, scratch wound healing assays in vitro, and corneal epithelial wound healing assays in vivo to determine the role of the AQP3/PLD2/PG signaling pathway in corneal epithelium.

**Results:**

AQP3 was present in human corneas in situ, and AQP3 and PLD2 were co-immunoprecipitated from corneal epithelial cell lysates. The two proteins could also be co-immunoprecipitated from insect cells simultaneously infected with AQP3- and PLD2-expressing baculoviruses, suggesting a likely direct interaction. A particular PG, dioleoylphosphatidylglycerol (DOPG), enhanced scratch wound healing of a corneal epithelial monolayer in vitro. DOPG also accelerated corneal epithelial wound healing in vivo, both in wild-type mice and in a mouse model exhibiting impaired corneal wound healing (AQP3 knockout mice).

**Conclusions:**

These results indicate the importance of the AQP3/PLD2/PG signaling pathway in corneal epithelial cells and suggest the possibility of developing DOPG as a pharmacologic therapy to enhance corneal wound healing in patients.

Blindness from corneal etiologies is a serious global issue limiting the productivity and quality of life of approximately 4.9 million people around the world.^1^ The cornea, an avascular, transparent tissue, allows the entry and initial focusing of light in the visual system and indeed is the most powerful refractive element in the eye. An irregular corneal surface results in substantial degradation of optical acuity. The stratified squamous epithelium, the outermost layer of the cornea, is integral in maintaining optical clarity and defending against microbial infection of the eye. The corneal barrier can be breached as a result of injuries, such as corneal abrasions or chemical burns, as well as several eye disorders including recurrent corneal erosions and Stevens–Johnson syndrome. Wounds can also be caused by corneal surgeries to correct vision, such as cataract removal, corneal transplantation, and refractive surgical procedures (e.g., laser in situ keratomileusis). Although corneal wounds generally heal rapidly, in a subset of patients these wounds heal slowly (so-called refractory corneal wounds) or recur, particularly in patients with other comorbidities such as diabetes.^2^^–^^4^ In addition, the pain associated with corneal injuries and the possibility of infection due to the disrupted barrier make the discovery of agents to accelerate corneal wound healing highly desirable.

Although there are clear differences between corneal epithelial cells and keratinocytes comprising the skin epithelium (the epidermis), these two cell types also exhibit similarities: both cells form stratified epithelia exposed to the environment and express many of the same genes/proteins, including immature keratin-14^5^; the differentiation markers, involucrin, loricrin, and transglutaminase^6^^,^^7^; and the water/glycerol channel aquaporin-3 (AQP3).^8^^,^^9^ In addition, elevated extracellular calcium concentrations inhibit the proliferation of both cell types,^10^ which can be grown in vitro using the same culture medium.^6^ Both cell types exhibit a programmed pattern of differentiation that includes the expression of mature keratins (keratins-1 and -10 in the epidermis and keratins-3 and -12 in the cornea).^10^^,^^11^ Furthermore, both cell types exhibit a migratory phenotype that is induced by epithelial wounding.^12^ Finally, corneal epithelium can transdifferentiate into a hair-bearing epidermis under the appropriate conditions.^13^^,^^14^ Therefore, we hypothesized that the function of these cells might be regulated by similar mechanisms.

In previous studies of epidermal keratinocytes, we have shown that the aquaglyceroporin AQP3 physically and functionally associates with the lipid-metabolizing enzyme phospholipase D2 (PLD2)^15^^,^^16^ such that glycerol transported into the cell by AQP3 is converted by PLD2 to phosphatidylglycerol (PG).^16^ Furthermore, manipulating this novel AQP3/PLD2 signaling module to alter PG production regulates keratinocyte proliferation.^17^ Indeed, direct treatment of keratinocytes with PG derived from egg results in an inhibition of keratinocyte proliferation in rapidly dividing keratinocytes^17^; however, in slowly dividing cells egg PG stimulates proliferation.^17^ Egg PG also accelerates skin wound healing in vivo.^18^ Whether or not corneal epithelial cells also possess this AQP3/PLD2 signaling module, as well as the possible role of PG in corneal epithelial wound healing, is unknown.

Egg PG is a mixture of different PG species composed of various fatty acids. We determined that different PG species are more effective at promoting versus inhibiting keratinocyte proliferation. Thus, PG containing polyunsaturated fatty acids effectively inhibits rapidly dividing keratinocytes, whereas PG composed of monounsaturated fatty acids is efficacious in stimulating the growth of slowly proliferating keratinocytes.^19^ In particular, dioleoylphosphatidylglycerol (DOPG) was able to significantly enhance proliferation in keratinocyte cultures with low growth rates.^19^ Because cell growth is essential for wound healing, we hypothesized that DOPG might also accelerate corneal wound healing. In the experiments reported here, we investigated the AQP3/PLD2 signaling module in corneal epithelial cells and scratch wound healing in vitro, as well as the ability of DOPG to accelerate corneal epithelial wound healing in vivo.

## Materials and Methods

### Immunohistochemistry

Formalin-fixed human corneas were obtained from the Georgia Eye Bank (Atlanta, GA, USA). Paraffin-embedded specimens were sectioned, subjected to antigen retrieval, and stained as described in Voss et al.^20^ The antibody recognizing AQP3 (Alomone Labs, Jerusalem, Israel) was previously validated in our laboratory using western analysis and by demonstrating increased AQP3 immunoreactivity in the skin of an epidermal-targeted AQP3-overexpressing transgenic mouse model.^15^^,^^21^

### Immunofluorescence Microscopy

For immunofluorescent histochemistry, 5-µm sections were cut from formalin-fixed paraffin-embedded human corneal samples obtained from the Georgia Eye Bank. After paraffin removal, rehydration, and antigen retrieval, sections were stained using an Opal immunofluorescence kit (PerkinElmer, Inc., Waltham, MA, USA) following incubation with a rabbit polyclonal antibody recognizing PLD2 (Cell Signaling Technology, Danvers, MA, USA) and a mouse monoclonal antibody against AQP3 (Santa Cruz Biotechnology, Santa Cruz, CA, USA). Sections were then processed according to the supplier's instructions, and staining was visualized using confocal microscopy on a Zeiss laser-scanning microscope (Carl Zeiss Meditec, Jena, Germany). Immunofluorescent cytochemistry was performed on paraformaldehyde-fixed, Triton X-100-permeabilized immortalized human corneal limbal epithelial cells (Sigma-Aldrich, St. Louis, MO, USA), as described in Zheng et al.,^15^ using antibodies recognizing AQP3 and PLD2 and visualized using an Opal immunofluorescence kit as above.

### Cell Culture

Immortalized human corneal epithelial (IHCE) cells were a generous gift of Fu-shin Yu (Wayne State University, Detroit, MI, USA). This cell line represents an SV40-immortalized but otherwise normal human corneal epithelial cell line as described in Araki-Sasaki et al.^22^ These cells were cultured in a 1:1 mixture of defined keratinocyte serum-free medium (Gibco; Thermo Fisher Scientific, Waltham, MA, USA) and minimum essential medium (Cellgro; Corning Inc., Corning, NY, USA) with medium replacement performed approximately every 2 days. For treatment with phospholipid, PG in chloroform was aliquoted and the organic solvent evaporated under nitrogen. For application to cells or corneas, PG was hydrated in an aqueous solution and sonicated to form liposomes. Phospholipid liposomes, which are routinely used as drug delivery vehicles, are thought to deliver their contents to cells through fusion with the plasma membrane and/or endocytosis.^23^ In either case, this process should also result in internalization of the phospholipids comprising the liposomes and/or their incorporation into the plasma membrane. The telomerase-immortalized human corneal limbal epithelial cell line was obtained from Ilene Gipson (Harvard Medical School, Boston, MA, USA) and cultured as described previously.^24^

### Co-Immunoprecipitation

Cells were harvested using radioimmunoprecipitation assay (RIPA) buffer and immunoprecipitated using an antibody recognizing AQP3 (Alomone Labs) as described in Zheng et al.^15^ Immunoprecipitates were collected, solubilized in Laemmli buffer^25^ and 30 µg protein (determined using a BioRad protein assay kit with bovine serum albumin as the standard), and analyzed by western blotting, as described below.

### Western Analysis

Proteins in Laemmli buffer were separated by sodium dodecyl sulfate polyacrylamide gel electrophoresis on 8% gels as described in Zheng et al.^15^ After transfer of the separated proteins to Immobilon-P PVDF (MilliporeSigma, Burlington, MA, USA), immunoreactive bands were visualized using the appropriate primary and secondary antibodies. Bands were visualized using enhanced chemifluorescence (Amersham International, Little Chalfont, UK) and a Storm PhosphorImager (Molecular Dynamics, Sunnyvale, CA, USA).

### Protein Expression in Sf9 Insect Cells

Full-length mouse PLD2 bacmid, a generous gift of Guangwei Du **(**University of Texas Health Science Center, Houston, TX, USA), was cloned in pDEST 10. To clone AQP3 for insertion into the baculovirus vector, total mouse RNA was isolated from primary mouse keratinocytes using Trizol reagent (Life Technologies, Gaithersburg, MD, USA). Mouse cDNA was synthesized with the ThermoScript RT-PCR System (Invitrogen, Carlsbad, CA, USA) according to the manufacturer's instructions. The blunt-end PCR product of AQP3 (forward primer: 5′-CAC CAT GGG TCG ACA GAA GGA GTT GAT GAA TCG T-3′; reverse primers: 5′-TCA GAT CTG CTC CTT GTG TTT CAT GTG GGC-3′) was synthesized using Platinum *Pfx* DNA Polymerase (Invitrogen), verified by sequencing and cloned into pENTR/D-TOPO vector (Thermo Fisher Scientific) according to the manufacturer's instructions. An AQP3 entry clone was cloned into pDEST 20 using Invitrogen Gateway technology. Bacmids were made using the Bac-to-Bac Baculovirus Expression System (Thermo Fisher Scientific) according to the manufacturer's protocol. Sf9 cells were transfected with different bacmid constructs using Cellfectin Reagent II (Thermo Fisher Scientific), followed by virus amplification and titering according to the supplier's instructions.

Sf9 cells were infected with the baculovirus expressing AQP3, PLD2, or both for 48 hours in Sf9 complete medium. Cells were then lysed in RIPA buffer and homogenized. The supernatants were precleared with sheep anti-mouse or sheep anti-rabbit IgG-coated Dynal beads (Invitrogen Dynal AS, Oslo, Norway). The cleared samples were incubated with anti-PLD2, anti-His, anti-GST (1 µg/mL) or control serum for 2 hours at 4°C, followed by the addition of 40 µL sheep anti-rabbit or sheep anti-mouse IgG-coated Dynal beads for 1 hour at 4°C. Immunocomplexes were collected using magnetic separation, washed four times with RIPA buffer, solubilized in lysis buffer, and denatured at 80°C for 5 minutes, and equal volumes were subjected to western blot analysis. The blots were incubated with anti-PLD2, anti-His, or anti-GST antibody; washed extensively; and incubated with goat anti-mouse or goat anti-rabbit antibodies prior to visualization using enhanced chemifluorescence.

### Scratch Wound Healing Assay

IHCE cells were grown to confluence, and a 20- to 200-µL (yellow) pipet tip was used to make a scratch wound in the monolayer. Scraped cells were removed by rinsing with PBS, and the cultures were photographed at time 0 using an EVOS XL transmitted light microscope system (Thermo Fisher Scientific). Cells were then incubated in medium lacking added epidermal growth factor (EGF; except when the effect of EGF was tested) for ∼22 hours in control medium or medium containing the appropriate agents. Wounds were again photographed and the distance between the leading edges of the wound measured and subtracted from the wound size at time 0 to obtain a value for the difference in wound size.

### Corneal Wound Healing In Vivo

We investigated corneal epithelial wound healing in a mouse model of delayed wound healing (AQP3 knockout mice on a CD-1 strain background),^12^ as well as in wild-type, outbred CD-1 mice (to better model the heterogeneous human population). In the AQP3 knockout mice, corneal wounds were made and wound healing was followed over time in animals treated with PBS control vehicle (Con) or 250 µg/mL DOPG in PBS. Approximately 10 µL of the PBS control vehicle or DOPG in PBS was added dropwise at the indicated time points. This particular dose was selected based on the in vitro results shown in Figure 5 and their biphasic nature, such that a higher dose produces less of a response. On the other hand, the expected rapid clearance of the solution from the eye surface would result in minimal contact with the corneal cells as a result of a robust blinking response; therefore, the tested concentration was increased approximately 10-fold. In addition, it was thought that, due to the lack of the AQP3 to transport the PG precursor glycerol, the AQP3 knockout mouse cornea might be deficient in PG. Therefore, for CD-1 mouse experiments, a lower dose of 100 µg/mL DOPG in PBS was used because the presence of the complete AQP3/PLD2 signaling module in wild-type corneal epithelial cells suggested that CD-1 mouse corneas might have higher PG levels than the AQP3 knockout mice lacking a key component of this module. Wound healing studies were performed using the scrape wound model as in Elizondo et al.,^26^ and all procedures were approved by the Augusta University Institutional Animal Care and Use Committee (protocol no. 2013-0581) in accordance with the ARVO Statement for the Use of Animals in Ophthalmic and Vision Research.

Briefly, mice were anesthetized with ketamine/xylazine (80/6 mg/kg body weight), and fluorescein (Altafluor Benox, a drop; Altaire Pharmaceuticals, Aquebogue, NY, USA) was applied to the eye. A corneal wound covering ∼70% of the corneal surface was then generated with an Algerbrush, taking care to spare the limbal region. The wound was photographed, and PBS with or without DOPG was applied. Every ∼4 hours as indicated, mice were anesthetized with isoflurane and the wounds photographed after fluorescein application. At this time, the wound was also re-treated with PBS with or without DOPG. The wound margin was traced, the area quantified using ImageJ software (National Institutes of Health, Bethesda, MD, USA), and the results were expressed as means ± SEM relative to the wound area at time 0. Slopes were determined by linear regression of the measured values until complete closure of the corneal wound as in Elizondo et al.^26^

### Statistics

Statistical analysis on three or more groups was performed using ANOVA followed by Student–Newman–Keuls post hoc tests; a Student's *t*-test was used to determine the significance between two groups. Analyses were performed using Prism or InStat software (GraphPad, La Jolla, CA), with significance established at *P* ≤ 0.05.

## Results

### AQP3 Protein is Expressed in Human Cornea In Situ

**Figure 1.**
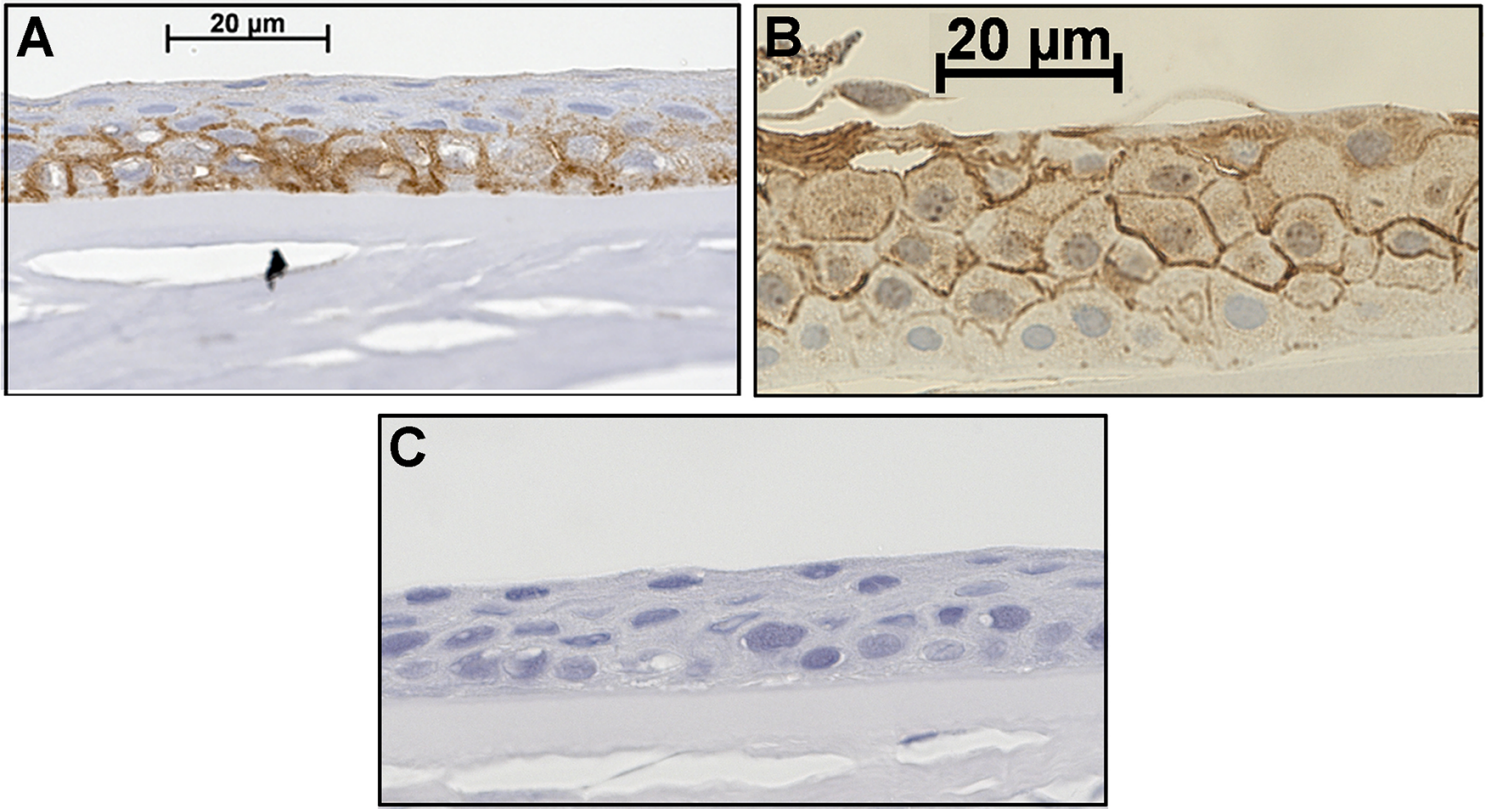
Human corneal epithelial cells express AQP3 in situ. Formalin-fixed human corneas obtained from the Georgia Eye Bank were paraffin-embedded and sectioned. After antigen retrieval, sections were incubated with an antibody recognizing AQP3 and visualized with an ABC staining kit using 3,3′-diaminobenzidine as chromogen (brown staining), as described in the Materials and Methods section. Sections were counterstained with hematoxylin (blue staining) and photographed. Results are representative of the staining of random sections of right and left corneas from at least three individuals. (**A**, **B**) AQP3 staining observed in two different corneas. (**C**) A negative control performed by omission of the primary antibody.

**Figure 2.**
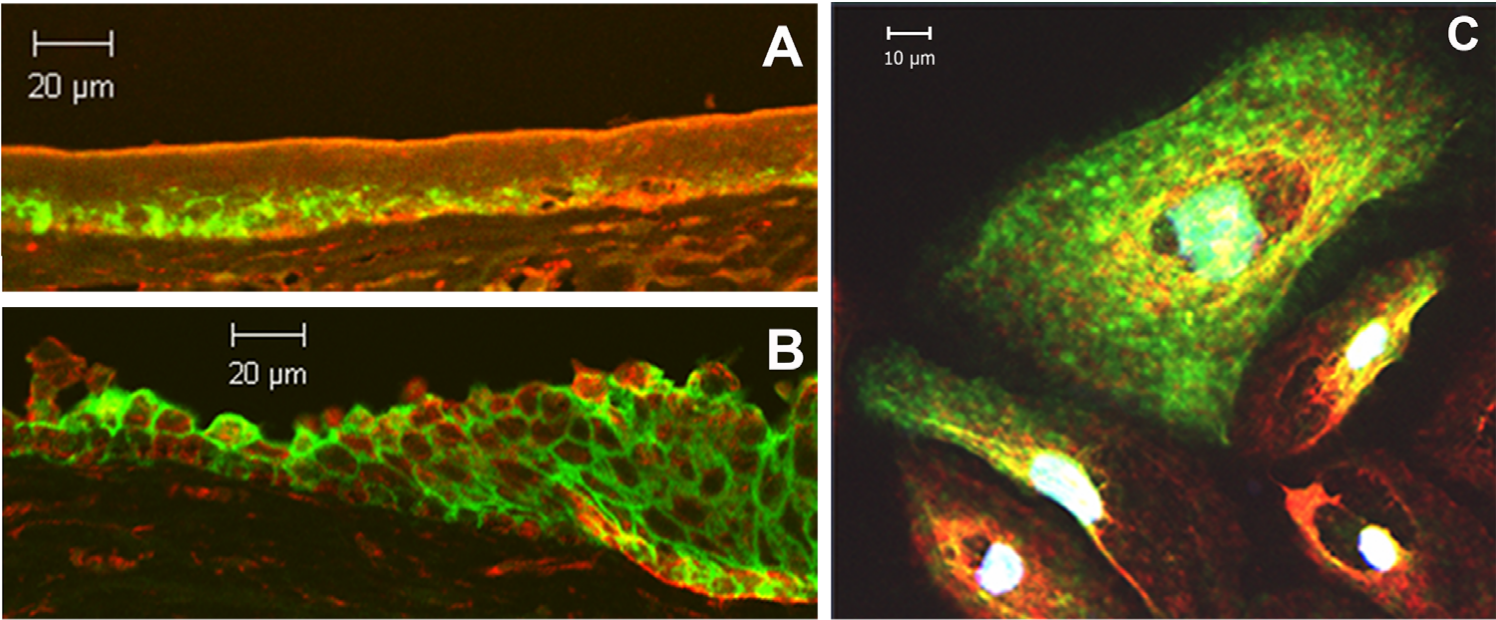
AQP3 and PLD2 co-localize in the cornea in situ and in immortalized human corneal limbal epithelial cells in vitro. Formalin-fixed human corneas obtained from the Georgia Eye Bank were paraffin-embedded and sectioned onto microscope slides. Sections were stained using an Opal immunofluorescence kit and incubated with a rabbit polyclonal antibody recognizing PLD2 and a mouse monoclonal antibody against AQP3. Sections were then processed according to the supplier's instructions with red staining (Opal 570) representing PLD2 and green (Opal 520) representing AQP3. Staining was visualized using confocal microscopy on a Zeiss laser-scanning microscope. (**A**) A more central region of the corneal epithelium, (**B**) the limbal region of the cornea, and (**C**) immortalized human corneal limbal epithelial cells are shown. Results are representative of at least three corneas or cell passages; negative controls in which primary antibodies were omitted demonstrated essentially no staining.

Human corneas showed AQP3 immunoreactivity that localized primarily to corneal epithelial cell borders (Fig. 1). Two patterns were observed: (1) staining observed primarily in the basal and early differentiated layers with little observed in more superficial layers (Fig. 1A), and (2) AQP3 immunoreactivity in the differentiated upper layers with sparse staining in the basal layer (Fig. 1B). Nevertheless, AQP3 protein was clearly present in epithelial cells of the cornea. A negative control in which the primary antibody was omitted demonstrated no non-specific staining (Fig. 1C).

### AQP3 and PLD2 Partially Co-localize in Human Corneal Limbal Epithelial Cells and Human Cornea

To determine if AQP3 and PLD2 co-localize in the human cornea, we performed immunofluorescent confocal microscopy on human corneal limbal cells and human corneas. We found that AQP3 and PLD2 partially co-localize in cornea in situ. Interestingly, we observed a greater degree of co-localization in the limbal region (Fig. 2B) than in the central cornea (Fig. 2A), consistent with a possible role of this pathway in corneal wound healing. We also demonstrated partial co-localization of AQP3 and PLD2 in human corneal limbal cells (Fig. 2C), similar to our findings in epidermal keratinocytes.^15^

### AQP3 and PLD2 Co-immunoprecipitate from Immortalized Human Corneal Epithelial Cells

**Figure 3.**
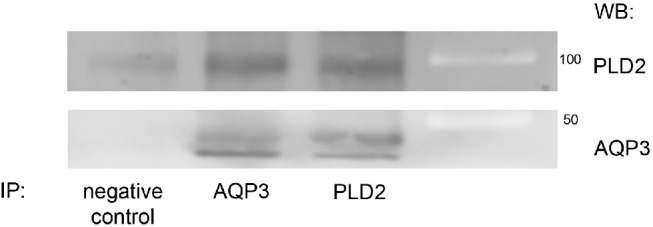
Immortalized human corneal epithelial cells express both AQP3 and PLD2 proteins, which can be co-immunoprecipitated from these cells. SV40-immortalized human corneal epithelial cells were cultured in a 1:1 mixture of defined keratinocyte serum-free (DKSF) medium and Minimum Essential Medium until approximately 70% confluent. The cells were harvested using RIPA buffer and immunoprecipitated using antibody recognizing AQP3 as described in the Materials and Methods section. Immunoprecipitates were collected and analyzed by western blotting with antibodies recognizing AQP3 or PLD2 as indicated. Molecular weight standards were separated in the far right lane and appear as light bands. Results are representative of at least three separate experiments.

To determine whether AQP3 associates with PLD2 in corneal epithelial cells, we examined whether PLD2 and AQP3 could be co-immunoprecipitated from IHCE lysates. Figure 3 demonstrates that AQP3 (both glycosylated and unglycosylated forms) co-immunoprecipitates with PLD2 and vice versa.

### The Association of AQP3 with PLD2 is Likely Direct

**Figure 4.**
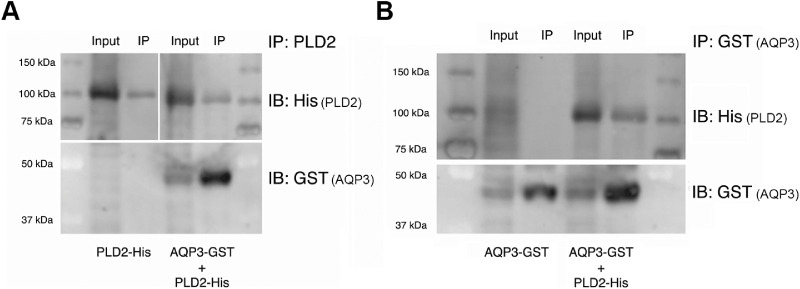
The interaction between PLD2 and AQP3 is likely direct. Sf9 insect cells were infected with baculovirus expressing either His-tagged PLD2 (PLD2-His; Fig. 4A, left panel) or GST-tagged AQP3 (AQP3-GST; Fig. 4B) alone or with both AQP3-GST and PLD2-His (Fig. 4A, right panel, Fig. 4B). Equal volumes of pre-cleared Sf9 lysates were then immunoprecipitated (IP) with anti-GST antibody (to pull down AQP3-GST) or anti-PLD2 antibody, and the immunoprecipitates were analyzed by sodium dodecyl sulfate polyacrylamide gel electrophoresis (SDS-PAGE) and immunoblotting (IB) using antibodies recognizing GST (for AQP3-GST) or His (for PLD2-His) as indicated. A 1/10 volume of lysate was similarly analyzed (Input). Results are representative of three experiments.

Our demonstration that AQP3 and PLD2 could be co-immunoprecipitated indicated a physical association but did not indicate whether this linkage was via direct binding or indirectly resulted from the formation of a complex with an adaptor or scaffolding protein. Therefore, to determine if the interaction between AQP3 and PLD2 was direct or via an adapter or scaffolding protein, we instead used an evolutionarily more distant expression system: Sf9 insect cells. Thus, we expressed His-tagged PLD2 and glutathione *S*-transferase (GST)-tagged AQP3 in an Sf9 insect cell expression system and performed co-immunoprecipitation assays. As shown in Figure 4, immunoprecipitation of AQP3 with an anti-GST antibody resulted in co-immunoprecipitation of PLD2, and immunoprecipitated PLD2 co-immunoprecipitated AQP3, but only when the two baculoviruses were both used to infect the Sf9 cells. These results suggest that the interaction between AQP3 and PLD2 is direct, as it seems unlikely that insect cells would express an adapter or scaffolding protein with sufficient homology to the mammalian protein to allow its interaction with, and co-immunoprecipitation of, AQP3 and PLD2.

### DOPG Enhances Scratch Wound Healing of Immortalized Corneal Epithelial Cells In Vitro

**Figure 5.**
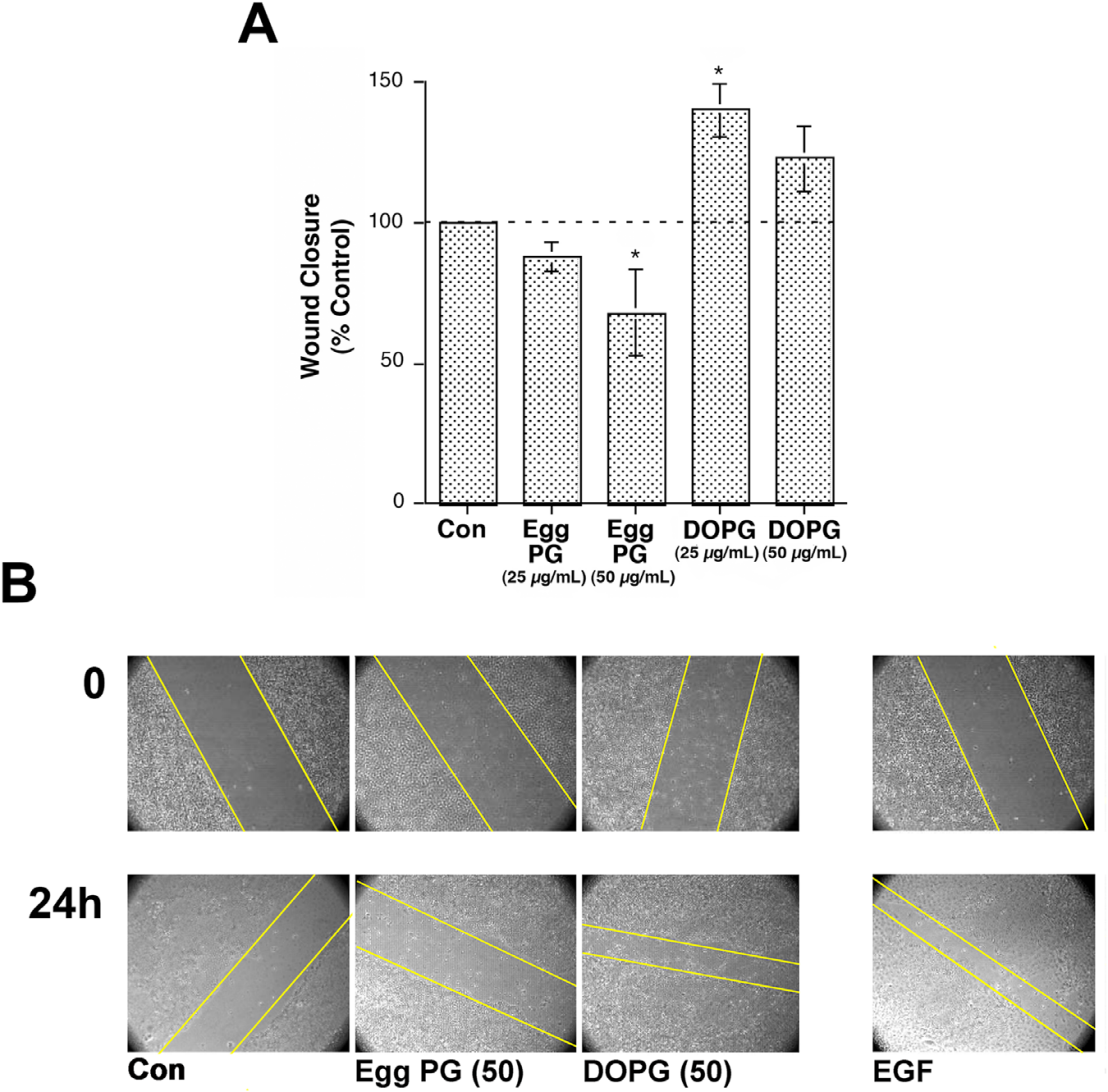
DOPG stimulates, whereas egg PG inhibits, scratch wound healing of immortalized human corneal epithelial cells. Immortalized human corneal epithelial cells were grown to confluence, and a pipet tip was used to make a scratch wound in the monolayer. Wounds were photographed at time 0 and at various time points thereafter. (**A**) The distance between the leading edges of the wound was determined at 22 hours and subtracted from the wound size at time 0 to obtain a value for the difference in wound size. These differences were then expressed relative to the difference observed in control (untreated) cells (100%). Results represent the means ± SEM of three separate experiments (^*^P < 0.05 relative to the control using ANOVA and a Newmann–Keuls post hoc test). (**B**) A representative experiment showing wound closure over time; the approximate wound edges are indicated with yellow lines.

Based on the finding that the PG species DOPG is most effective in stimulating keratinocyte proliferation,^19^ we investigated the effect of DOPG versus PG, which was derived from egg as a control, on corneal epithelial cell scratch wound healing in vitro. Figure 5 shows that, in IHCE cells, DOPG stimulated scratch wound healing by approximately 40%. This stimulation was comparable to the effect of a positive control, epidermal growth factor (5 ng/mL), which increased scratch wound closure by 61 ± 22% relative to the control (data not shown). On the other hand, egg-derived PG, composed of a mixture of fatty acids (as discussed in Xie et al.^19^), actually inhibited scratch wound healing by approximately 30% (Fig. 5), indicating the specificity of the effect of DOPG on corneal epithelial wound healing in vitro.

### DOPG Accelerates Corneal Epithelial Wound Healing in AQP3 Knockout Mice In Vivo

**Figure 6.**
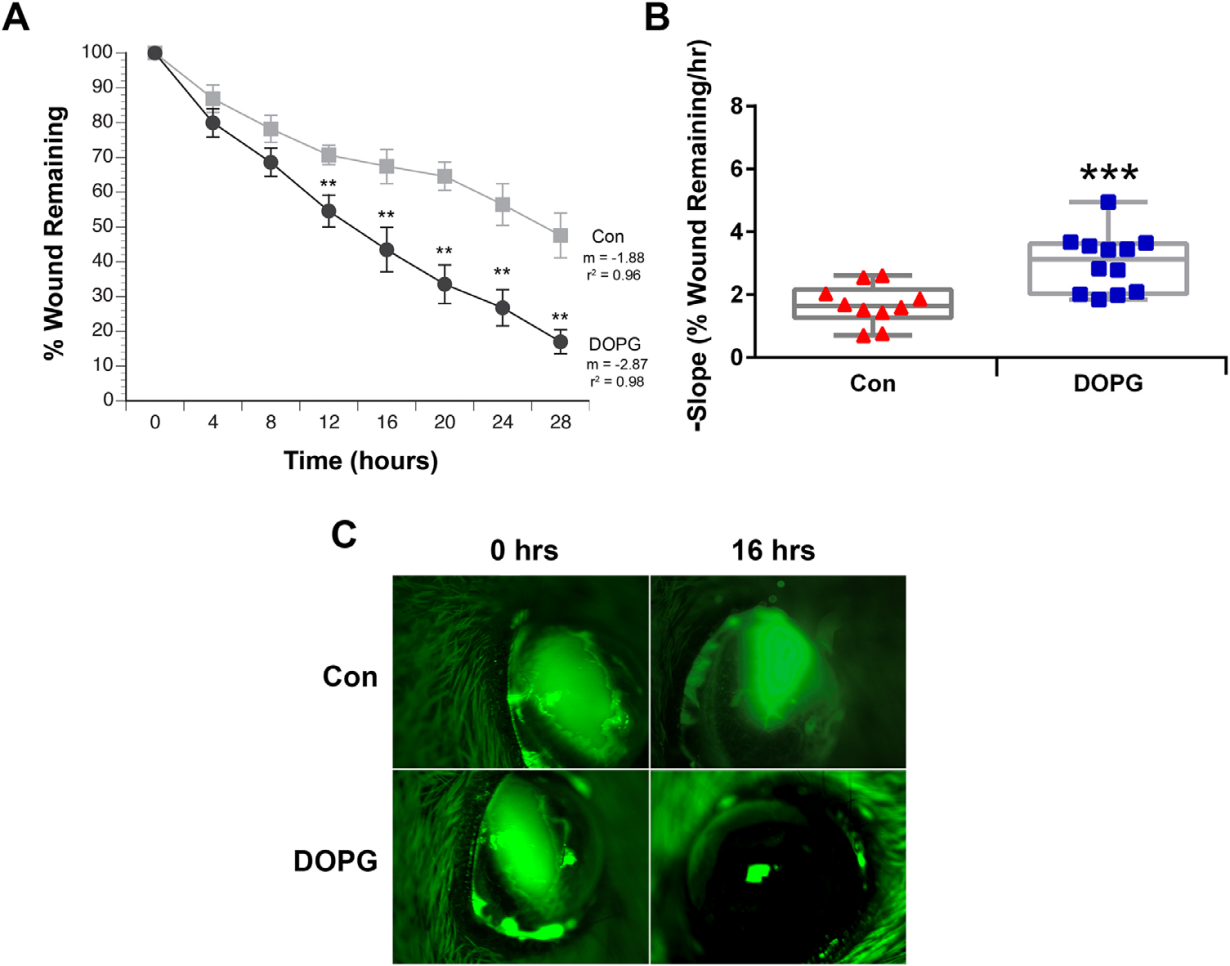
DOPG accelerates corneal epithelial wound healing in AQP3 knockout mice. Anesthetized mice (n = 10–12) received corneal wounds produced by scraping the epithelium with an Algerbrush. Fluorescein was added to the wounded eye, and the wound was photographed. Saline control vehicle (Con) or DOPG in saline (250 µg/mL) was then applied to the eye. This process was repeated every 4 hours for 28 hours. The area of the photographed wounds was digitized and expressed as a percentage of the initial wound area. (**A**) Mean ± SEM of the wound area at each Con versus DOPG time point; these values were compared using an unpaired Student's t-test, with ^**^P < 0.01 versus Con at the corresponding time point. (Please note that, by ANOVA followed by a Student–Newman–Keuls post hoc test, all values for both Con and DOPG were significantly different from time zero.) Wound healing rates were calculated by performing linear regression on the data for each wound until complete closure. (**B**) Regression slopes (the healing rate) were also compared by an unpaired Student's t-test and illustrated as the values for individual mice (^***^P < 0.01 vs. the Con mean value). (**C**) Illustrated are representative corneal wounds at 0 and 16 hours.

**Figure 7.**
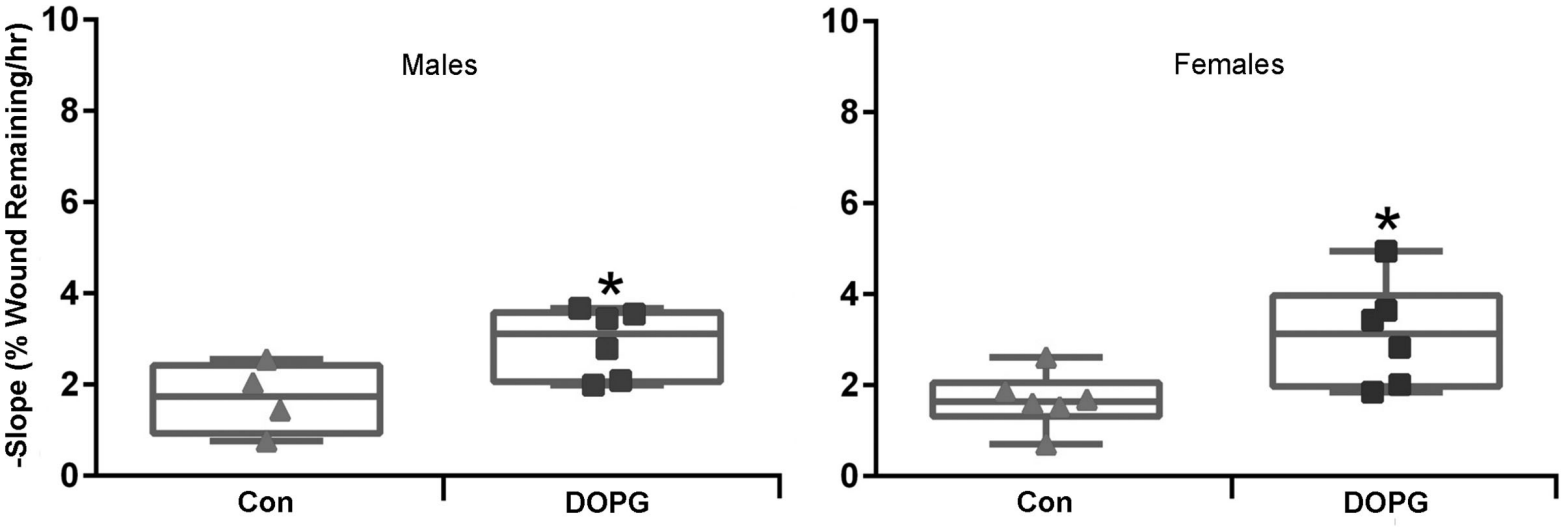
The ability of DOPG to enhance corneal epithelial wound healing is not affected by the sex of the animal. Male and female AQP3 knockout mice treated as in Figure 6 were analyzed separately (n = 4–6). Regression slopes (the healing rate) were compared by an unpaired Student's *t*-test and illustrated as values for individual animals by sex as indicated (^*^P < 0.05 vs. the Con mean value).

Our results in corneal epithelial cells suggested the possibility that DOPG might be able to stimulate corneal epithelial wound healing in vivo. To examine this idea, we first investigated corneal epithelial wound healing in a mouse model of impaired corneal epithelial wound healing, with the idea that positive effects might be more pronounced. Thus, we investigated corneal epithelial wound healing in AQP3 knockout mice, which have been shown previously to exhibit delayed wound healing.^12^ Healing was found to be accelerated by DOPG treatment. With time point comparisons, significant differences in healing between control vehicle- and DOPG-treated animals were found at each time point 12 hours and beyond after wounding (Fig. 6). In fact, the extent of improvement seen after DOPG treatment is remarkable. For example, at the 28-hour final time point, wounds were on the order of 50% smaller in DOPG-treated corneas versus vehicle-treated controls. In addition, healing rates (slopes) were significantly different (*P* < 0.001). To determine whether a sex difference affected the response to DOPG, both male and female mice were used. When analyzed separately, we found that there was no difference in the ability of DOPG to accelerate healing in males versus females (Fig. 7).

### DOPG Accelerates Corneal Epithelial Wound Healing in Wild-Type Outbred Male Mice In Vivo

**Figure 8.**
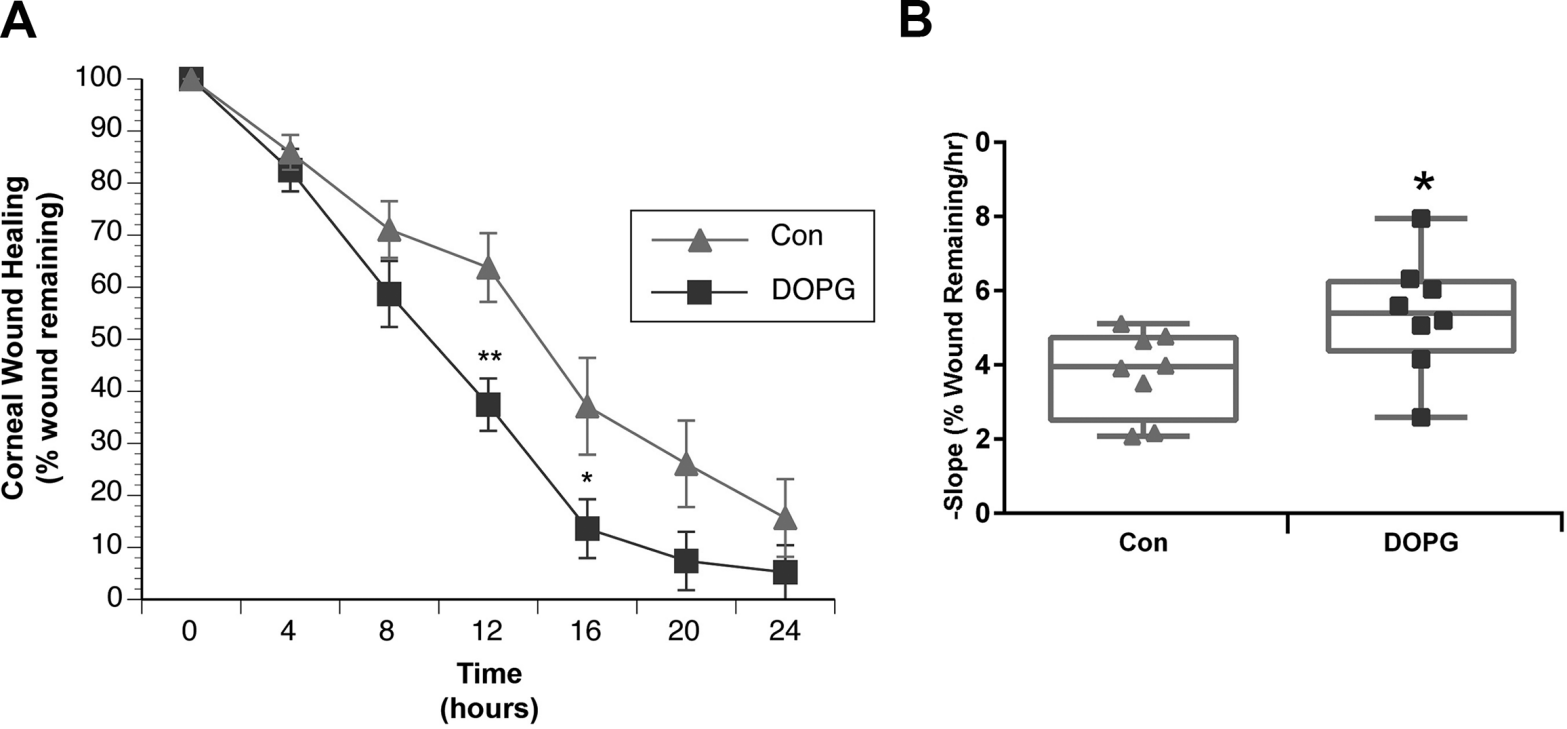
DOPG accelerates corneal epithelial wound healing in Institute of Cancer Research CD1 outbred mice. Anesthetized mice (n = 8) received corneal wounds as in Figure 6. Fluorescein was added to the wounded eye, which was photographed, and then saline control vehicle (Con) or DOPG in saline (100 µg/mL) was applied to the eye every 4 hours for 24 hours. The photographed wounds were analyzed as in Figure 6. (**A**) Illustrated are the means ± SEM of the wound area for Con versus DOPG at each time point; these values were compared using an unpaired Student's t-test, with ^*^P < 0.05 and ^**^P < 0.01 versus the corresponding Con time point. Wound healing rates for individual mice were calculated by performing linear regression on the data until complete wound closure. (**B**) Regression slopes (the healing rate) were also compared by an unpaired Student's t-test and are illustrated (^*^P < 0.05 vs. the Con mean value).

We then wished to determine if DOPG could enhance corneal epithelial wound healing in normal wild-type mice and investigated corneal epithelial wound healing in the CD-1 outbred mouse model (selected to more closely mimic the heterogeneity of the human population). Corneal wounds were created in the eyes of CD-1 male mice, and these mice were then treated with vehicle or DOPG (100 µg/mL) as described in the Materials and Methods section. DOPG significantly enhanced corneal wound healing, with significant differences found at the 12- and 16-hour time points (Fig. 8A). Linear regression calculation of the wound healing rates (negative slopes in Fig. 8B) showed that DOPG also significantly enhanced the rate of healing. This result indicates that DOPG is also able to accelerate corneal epithelial wound healing in normal outbred (heterogeneous) mice.

## Discussion

Our results indicate that DOPG can potently accelerate corneal epithelial wound healing in vitro and in vivo. DOPG accelerated scratch wound closure of immortalized corneal epithelial cells in culture by about 40%, comparable to the approximately 60% stimulation observed with the positive control, EGF. Furthermore, this effect was specific, as egg PG, a mixture of PG species, inhibited scratch wound healing. Interestingly, however, the response was biphasic, with a higher 50-µg/mL DOPG concentration resulting in a lower rate of wound healing than the 25-µg/mL dose. For the AQP3 knockout mice in vivo wound healing experiments, because rapid blinking tends to flush the applied solution from the eye and limits the exposure of the corneal epithelial cells to its components, we elected to use 250 µg/mL DOPG, or 10 times the closure-enhancing concentration in vitro. This dosing decision was also based in part on the idea that, in the AQP3 knockout mice, corneal PG levels might be expected to be deficient, as AQP3 would not be present to funnel glycerol to PLD2 for conversion to PG. This decision was also based on the fact that in wild-type mice, although 100 µg/mL DOPG (four times the best in vitro dose) significantly enhanced corneal epithelial wound healing, the effect was not marked, suggesting that the concentration might not be optimal. Indeed, despite the significant effects of DOPG to accelerate corneal epithelial wound healing observed in both cases, it seems possible that the doses selected for investigation still may not be optimal. However, we note that the lipid concentrations used (100 to 250 µg/mL) are comparable to those used for in vitro experiments examining DOPG activation of protein kinase C βII (PKCβII)^27^ for intact cell experiments in retinal pigment epithelial cells,^28^ keratinocytes,^19^ and alveolar macrophages,^29^^–^^32^ and for in vivo infection control in mouse lungs.^29^^,^^30^ In addition, greater lipid concentrations are used for surfactant supplementation in preterm infant lungs (for which 135 mg surfactant in 5 mL, or 27 mg/mL, is recommended^33^). Clearly, future investigations will be needed to determine the concentration of DOPG required for maximal stimulation of corneal epithelial wound healing.

We proposed that PG is produced by the combined action of AQP3 and PLD2, and, indeed, we demonstrated in keratinocytes that the increase in PG levels elicited by an elevated calcium concentration could be inhibited by ethanol,^16^ indicating mediation by PLD. We demonstrated that both AQP3 and PLD2 proteins are expressed in corneal epithelial cells and that both are also found in human corneas in situ. Furthermore, we demonstrated that AQP3 and PLD2 can be co-immunoprecipitated from corneal epithelial cells just as from epidermal keratinocytes^15^; therefore, it seems possible that PG is produced by this signaling module in the cornea as well as in the epidermis. Interestingly, the interaction between AQP3 and PLD2 appears to be direct rather than mediated by an adapter or scaffolding protein. We attempted to test this idea initially using an in vitro transcription-translation approach with a rabbit reticulocyte lysate system and AQP3 and PLD2 expression plasmids. Although under these conditions in vitro transcribed-translated AQP3 and PLD2 could be co-immunoprecipitated, we detected co-immunoprecipitation even if only one plasmid was included in the reaction (data not shown), likely because red blood cells express AQP3^34^^,^^35^ and PLD activity.^36^ However, if these cells express both AQP3 and PLD2, they would also probably express an adaptor or scaffolding protein, if the interaction were indirect. For this reason we repeated the co-immunoprecipitation studies using the Sf9 expression system. Based on the fact that the two proteins could be co-immunoprecipitated from Sf9 insect cells only when both AQP3 and PLD2 were expressed, our results suggest a direct interaction between AQP3 and PLD2, particularly because it seems unlikely that the insect cells would express a homolog of a mammalian adapter/scaffolding protein with a sufficient degree of similarity to allow co-immunoprecipitation of AQP3 and PLD2 in the absence of a direct association. A direct interaction between these two proteins suggests that it might be possible to disrupt the ability of AQP3 and PLD2 to associate and form PG, thereby possibly providing a mechanism to regulate corneal PG levels. Thus, future studies to determine the AQP3 and PLD2 interacting domains seem warranted.

The AQP3 knockout mice used in these studies are reportedly on the CD-1 strain background.^37^ These animals have been demonstrated to exhibit delayed corneal epithelial wound healing.^12^ Comparing the slopes of the AQP3 knockout versus the wild-type mice in Figures 6 and 8, it can be seen that the knockout mice showed slower rates of wound healing (lower slope values), and that 250 µg/mL DOPG stimulated this process to yield a rate comparable to that observed in wild-type animals. The ability of DOPG to enhance healing in a model of impaired corneal wound repair suggests the possibility that this agent might be useful in other eye conditions characterized by slow wound healing. Nevertheless, DOPG also accelerated corneal epithelial wound healing in wild-type animals, indicating its likely utility even in cases where wound healing is not delayed. Also of interest is the fact that the sex of the mouse does not influence the ability of DOPG to accelerate corneal epithelial wound healing, again suggesting the possible translational significance of these findings.

The mechanism by which DOPG enhances corneal epithelial wound healing is as yet unclear, but data in the literature suggest several possibilities. Fields and colleagues demonstrated that PG could bind to and stimulate the activity of the PKC isoform PKCβII over and above the activation observed with classical activators and that this activation mediates mitosis in human leukemia cells.^38^^,^^39^ In fact, PKCβII, which differs from its splice variant PKCβI in the carboxy terminus, was found to bind to PG via its final C-terminal 13 amino acids.^27^ In the above studies, DOPG was the most efficacious of the PG species tested,^39^ suggesting that DOPG might accelerate corneal epithelial wound healing by stimulating the activity of PKCβII. On the other hand, PG has also been shown to promote the assembly of proteins in cellular organelles such as thylakoid membranes^40^^,^^41^ and to restore membrane potential in depleted mitochondria.^42^ Chen et al.^43^ have shown that supplementation of a macrophage cell line with DOPG enhances mitochondrial function, which could increase the provision of ATP to fuel migration and proliferation. Emerging evidence also points to a potential role of PG in autophagy and mitophagy (reviewed in Hsu and Shi^44^). Finally, PG is known to inhibit toll-like receptor activation by microorganisms and microbial components, so-called pathogen-associated molecular patterns (PAMPs),^31^^,^^32^^,^^45^ as well as by danger- or damage-associated molecular patterns (DAMPs),^46^ endogenous molecules released by injured cells.^47^ Activated TLRs then induce an innate immune response including inflammation; therefore, it is possible that DOPG accelerates corneal wound healing by inhibiting inflammation. Because some inflammation may promote,^48^^,^^49^ whereas excessive inflammation inhibits,^48^ corneal healing, such a mechanism might also explain the observed biphasic effect of DOPG.

In summary, we have shown in corneal epithelial cells the presence of the AQP3/PLD2 signaling module, which in epidermal keratinocytes produces PG to regulate cell function.^15^^–^^17^^,^^19^ In addition, we demonstrate that a particular PG species, DOPG, which shows the greatest efficacy in stimulating the proliferation of slowly dividing epidermal keratinocytes,^19^ was able to enhance scratch wound healing of corneal epithelial cells in vitro as well as corneal epithelial wound healing in vivo. The capacity of DOPG to accelerate corneal epithelial wound healing was observed both in outbred (heterogeneous) wild-type mice and in a mouse model demonstrating delayed healing,^12^ AQP3 knockout mice. These results suggest the possibility of developing DOPG to enhance corneal epithelial wound healing in patients with corneal injuries or surgeries or in those with eye diseases characterized by impaired corneal wound healing. Commercialization of PG as a treatment for corneal epithelial wound healing seems particularly promising because (1) PG is a naturally occurring phospholipid, comprising approximately 10% of pulmonary surfactant lipids (reviewed in Veldhuizen et al.^50^) and found also in mitochondrial membranes (reviewed in Horvath and Daum^51^); (2) PG is already used as an inactive ingredient in ophthalmic agents, such as over-the-counter Systane (Alcon, Geneva, Switzerland) eye drops and Food and Drug Administration (FDA)-approved Visudyne (verteporfin; Bausch Health, Laval, Canada), a drug that is injected intravenously for the photodynamic treatment of age-related macular degeneration, among other eye diseases; and (3) PG is a component of FDA-approved drugs, such as Surfaxin (Discovery Laboratories, Warrington Township, PA, USA) for intratracheal administration in premature infants to reduce respiratory distress and improve breathing. Therefore, optimization of DOPG application protocols in preclinical mouse models should allow successful future administration of this agent to human patients with corneal wounds.
